# LEGO products have become more complex

**DOI:** 10.1371/journal.pone.0190651

**Published:** 2018-01-02

**Authors:** Christoph Bartneck, Elena Moltchanova

**Affiliations:** University of Canterbury, Private Bag 4800, 8140 Christchurch, New Zealand; Johns Hopkins University, UNITED STATES

## Abstract

The LEGO Group has become the largest toy company in the world and they can look back to a proud history of more than 50 years of producing bricks and other toys. Starting with a simple set of basic bricks their range of toys appeared to have increased in complexity over the years. We processed the inventories of most sets from 1955–2015 and our analysis showed that LEGO sets have become bigger, more colorful and more specialized. The vocabulary of bricks has increased significantly resulting in sets sharing fewer bricks. The increased complexity of LEGO sets and bricks enables skilled builders to design ever more amazing models but it may also overwhelm less skilled or younger builders.

## Introduction

The LEGO Group (TLG) has become the worlds largest toy company in the year 2015. The Danish brick-maker announced in its 2014 financial report [1] that revenues increased 11% in the first half of 2014. Total sales hit $2.03 billion USD, narrowly beating out Mattel’s $2 billion in revenue over the same period [2]. In particular The LEGO Movie and its franchise has been a key to their recent success. More recently the company lost 3% of its net profit and intends to lay off 1400 workers [3]. The rise of the company and the development of its products has been documented and discussed in detail [4, 5]. In particular the economic difficulties that nearly ruined TLG in the period leading up to 2004 and how the TLG recovered from it has been investigated [6]. The majority share of the popular LEGOLAND theme parks, for example, were sold to Merlin in 2005. The innovations that TLG produced resulted in a portfolio of products that go beyond the basic bricks. Today TLG also sells computer games, television series, books, clothing, and stationary. The LEGO themes are often associated to other media products, such as the Star Wars and Lord Of The Rings franchise. LEGO has been investigated as transmedial phenomenon [7] and even its connection to Philosophy has been explored [8].

The brick is no longer targeted exclusively at children. It is estimated that 5% of all LEGO purchases are made by adults that consider themselves as Adult Fans Of LEGO (AFOL). This group has dramatically grown in recent years which triggered the launch of several LEGO themed magazines, such as The Brick Journal, Blocks, and Bricks. The cult of LEGO has also been the topic of recent book releases [9].

Scientific investigations on the development of the LEGO products remain scarce. [10] showed that the faces of Minifigures has become more diverse and that the number of angry faces has increased. Their original data survey was the basis for the development of a pictorial scales for assessing affective responses [11].

At times quantitative studies are being performed to investigate common misconceptions about LEGO products. A typical misconception is that LEGO has become more expensive. [12] showed that LEGO sets have not become more expensive, they just contain more bricks. The average price per brick has actually slight decreased.

The interest in the development of the product prices is not limited to purchasing decision for the private use of LEGO sets, such as for birthday presents. Due to a growing secondary market for bricks and sets LEGO has become the object of financial investors. The Brick Picker (https://www.brickpicker.com/) website is dedicated to advising investors what sets to buy and when to sell. The most popular dedicated trading platforms for LEGO products is currently Bricklink.com and Brickowl.com. Many special LEGO sales can also be observed on the more general online platforms, such as eBay.

The goal of this study is to investigate further common perceptions of the LEGO bricks in particular from a historical perspective such as:

1. How many new bricks and sets did TLG introduce each year?
2. Have the LEGO products become more colorful?
3. Have LEGO bricks become more specialized?
  - Has the number of bricks that occur in only one set increased?
  - Do LEGO sets contain more specialized bricks?
4. Do LEGO sets share less bricks with other sets of the same year?
5. Did the number of sets that can be build by taking bricks from other sets decrease?

## Method

To address the research questions mentioned above we executed several statistical operations. Any of these statistical operations are only as good as the data upon they are used. There are some catalogs of LEGO sets available [13] but their data is not available in a machine readable format. We therefore extracted the data from the most complete and up to data web platform: Bricklink.com. By using a professional data harvesting tool we have been able to collect the inventory of 10953 sets ranging from the year 1955 to 2015. Bricklink’s data was originally sourced from previous LEGO fan projects, such as LDRAW and Peeron. Since then Bricklink claims ownership of the meta data, which remains a disputed position [14]. In any case, the meta data included the exact inventory of all sets including the color and quantity of each brick. After the harvesting was complete Bricklink blocked the IP address of the computer from which the harvesting was executed. Clearly they do not like to share their data, in particular in the presence of other LEGO marketplaces, such as Brickowl. Bricklink does, however, allow the download of a list of all sets and a list of all bricks, but their matching remains off limits. The temporal trends were estimated using the generalised linear models (GLM). The distributions comparison was done using Kolmogorov-Smirnov test.

## Data processing

Our analysis was restricted to the sets which contained at least one minifigure or brick piece and which did not contain any sub-sets. There were a total of 10953 such sets, of which 5384(49.2%) contained brick pieces only, 4896(44.7%) contained both brick pieces and minifigures, and the rest contained a combination of brick pieces (P), minifigures (M), booklets (B) and gears (G). There were a total of 7593 different minifigures in the dataset, 47375 and 27303 different brick pieces with and without taking the colour into account respectively, 318 booklets and 1049 gears. A total of 130 different colours have been used throughout the years. All the data processing and analysis has been done in R using the *data.table* package [15]. The data is available [16].

## Results

**Fig 1:**
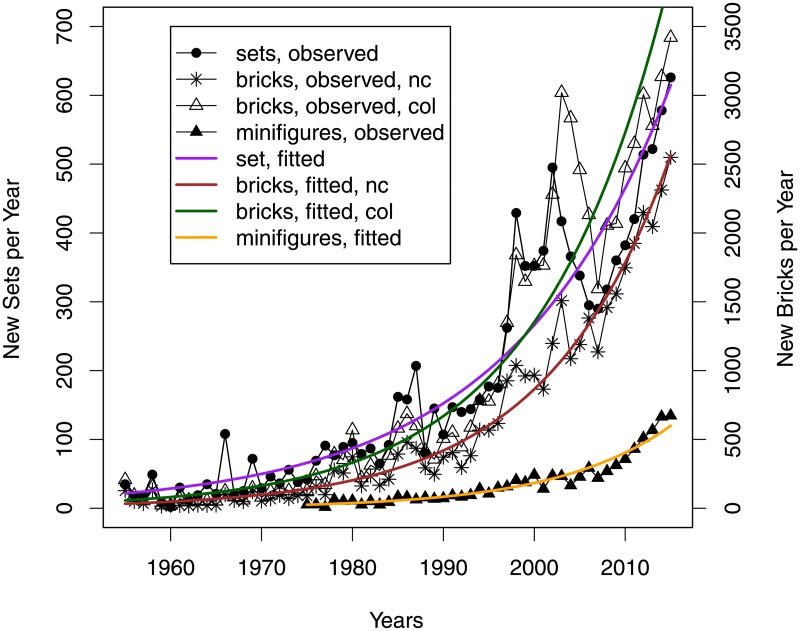
New sets and bricks released by year.

The trends in the number of annually released new sets and bricks (with or without taking the colour into account), and minifigures are shown in Fig 1. The numbers were found to increase annually by 5.7% (95% CI: 5.6%; 5.9%), 7.0% (95% CI: 6.9%; 7.1%), 6.9% (95% CI: 6.9%; 7.0%), and 8.2% (95% CI: 7.9%; 8.5%) respectively, (*p* < .0001).

**Fig 2:**
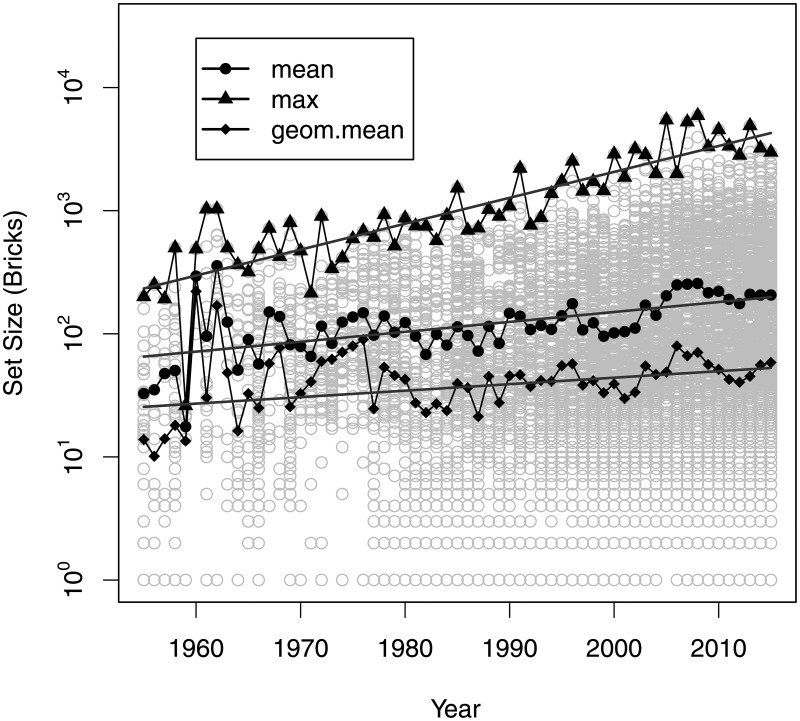
Set sizes by year.

The average size of a new set has been increasing by an average 1.9% per year (95% CI: 1.2%; 2.5%, *p* < .0001), and the size of the largest set has been increasing by an average 5.0% per year (95% CI: 4.2%; 5.8%, *p* < .0001), as shown in Fig 2. Furthermore, when comparing the size distributions by decades, we have found a clear shift towards larger sets. The p-values for the test of statistically significant difference in distributions between the decades 1976–1985 and 1986–1995, 1986–1995 and 1996–2005, 1996–2005 and 2006–2015 are *p* < .0001, *p* = 0.0022 and *p* = 0.01907 respectively. The number of colours in a set has been increasing at the average rate of 2.4% per year (95% CI: 2.3%; 2.5%, *p* < .0001), and the maximum number of colours in a set has been increasing at the average rate of 3.3% per year (95% CI: 3.1%; 3.5%, *p* < .0001).

While the sets were becoming larger, they were also becoming more diverse in terms of the number of different brick types involved. The average number of brick types in a set has been increasing at the average rate of 2.4% per year (95%*CI*: 2.2%; 2.6%, *p* < .0001), and the maximum number of brick types in a set has been increasing at the average rate of 4.1% per year (95%*CI*: 3.7%; 4.6%, *p* < .0001).

However, that also meant that the bricks were becoming more specialized. For example, the number of sets into which a brick was expected to be included in the next of 5 years, after adjusting for the total number of sets released in that period, was estimated to decrease annually by an average of 6.6% (95%*CI*: 6.5%; 6.7%) and 5.6% (95% CI: 5.5%; 5.7%) for the brick pieces with and without taking color into account respectively, and by an average of 6.6% (95% CI: 6.4%; 6.9%) for the minifigures (all *p* < .0001). There was also an apparent tendency for the brick to be included into less sets in the year of its release. However, this trend appears to have been reversed in 2006–2015, (*p* < .0001).

**Fig 3:**
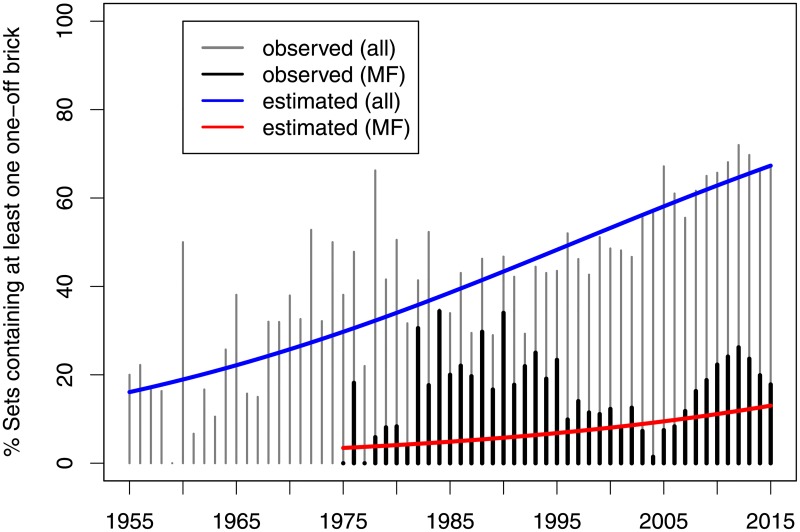
Proportion of sets, which have bricks used only once.

In general, 70.6% of all minifigures were found to be unique to a single set, which was significantly higher than the proportion of unique brick pieces, which was evaluated at 53.4% and 64.9% with and without taking color into account respectively. Over the entire study period, 2776 sets were found to contain at least one unique minifigure, 4789 sets were found to contain at least one unique brick (taking color into account). The odds of a set containing unique elements have steadily increased over the years at the average annual rate of 2.9% (95% CI: 2.6%; 3.3%) for colored bricks and that of 3.5% (95% CI: 2.8%; 4.4%) for minifigures (*p* < .0001) as shown in Fig 3. The number of unique bricks a set contained varied from 1 to 81 (the latter, a type-setters shop 9550-1).

### Color

**Fig 4:**
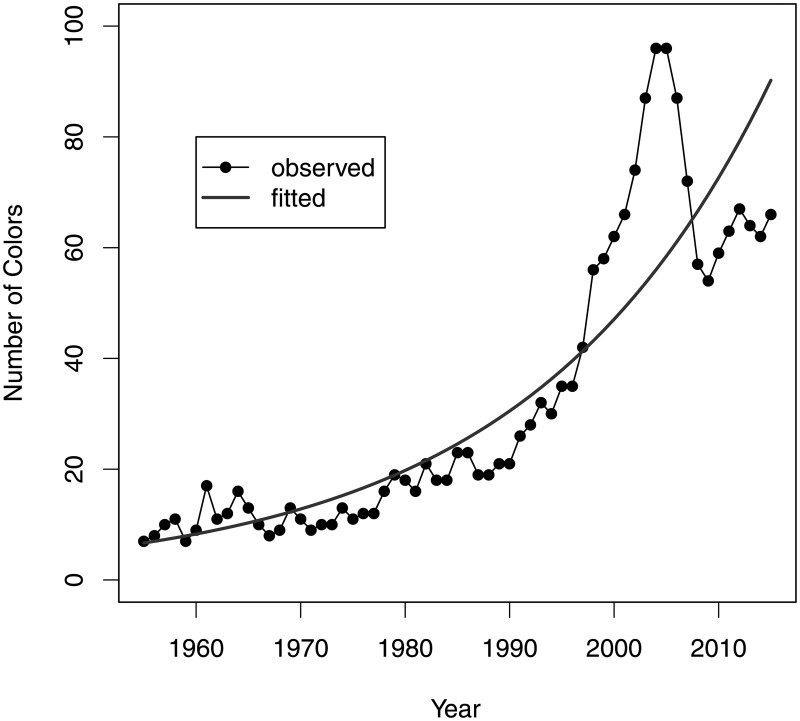
Colours used by year.

The number of colours used annually has also been found to increase exponentially at the average annual rate of 4.4% (95% CI: 4.1%; 4.7%, *p* < .0001) as shown in Fig 4. We have also evaluated Shannon’s entropy for the color distribution for each year, defined as

$$\begin{array}{c} S_{y}=-\sum_{c}p_{y}\left(c\right)\ln\left(p_{y}\left(c\right)\right), \end{array}$$ (1)

where *c* is the color index, *p*_*y*_(*c*) is the proportion of the brick pieces of color *c* used in the year *y*, and ln is the natural logarithm. We have also evaluated the Shannon’s entropy, using the proportion of the brick piece *types* rather than the overall quantities of color *c* used in the year *y*, but the results were very similar, so only the quantities-based Shannon’s entropy is shown in Fig 4. The entropy was found to have increased at the average annual rate of 1.1% (95% CI: 1.0%; 1.3%, *p* < .0001), confirming the impression that the sets are becoming more colorful with time.

**Fig 5:**
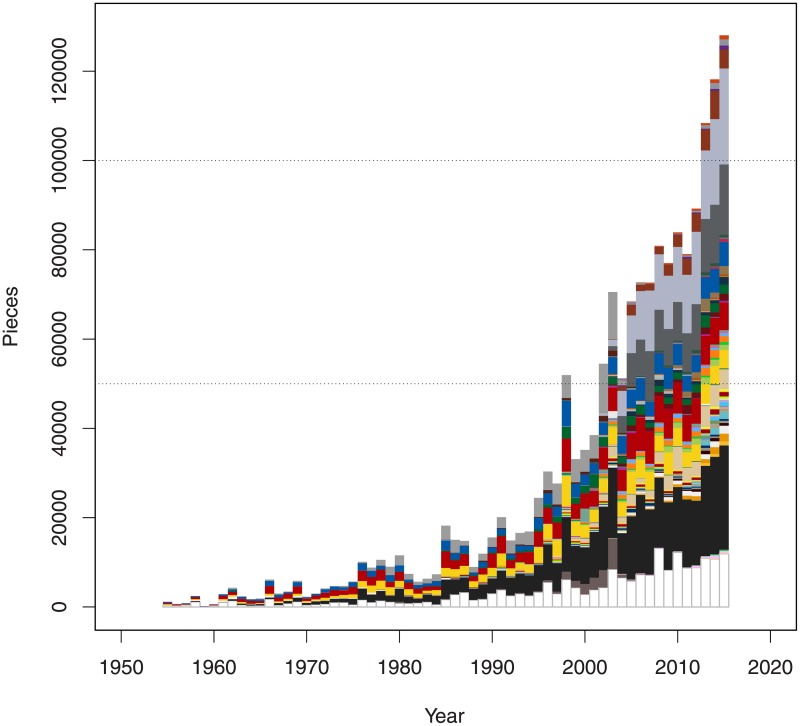
Frequencies of brick colors across time. The x-axis indicates years.

We plotted the number of bricks in each color across time (see Fig 5). Each pixel represents ten bricks. It is important to notice that besides solid colors, bricks also come in transparent and chrome colours, although they are dramatically less common.

**Fig 6:**
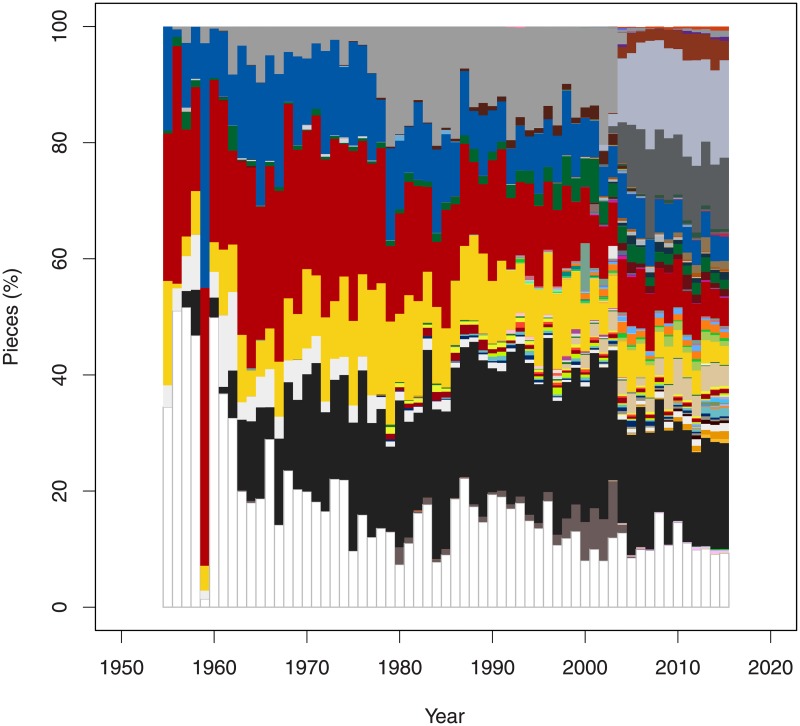
Relative frequencies of bricks colors across time. The x-axis indicates years.

Since the number of bricks available in each increased over the years it is difficult to understand how the colors used for each year have changed. We therefore calculated the relative proportion of each color for each years (see Fig 6).

While white, grey and black seem to have remained stable over the last centuries, the primary colors of red, yellow and blue have decreased. Many new colors and shades have emerged in particular in the last ten years.

It is also interesting to note the change in the two shades of grey in the year 2003. The new cooler greys completely replaced the old greys. A similar changed occurred for the dark brown which was replaced with a brighter more reddish brown.

Our results confirm the less formal analysis of [17]. A more detailed analysis of the LEGO colors is available [18]. He concluded that the exact definition of the colors that TLG is using is unclear and the colors used in this article can only be considered an approximate symbolic representation of the real LEGO colors.

### Commonalities

We first have to introduce a measure of commonality of any two sets, *i* and *j*, as follows:

$$\begin{array}{c} C_{i,j}=\frac{x_{ij}}{\min(n_{i},n_{j})}, \end{array}$$

where *n*_*i*_ and *n*_*j*_ are the respective sizes of the two sets, and *x*_*ij*_ is the number of bricks they have in common. The index is thus closer to 1 the more bricks are shared. If one set is completely included in the other one, the index *C* = 1.

**Fig 7:**
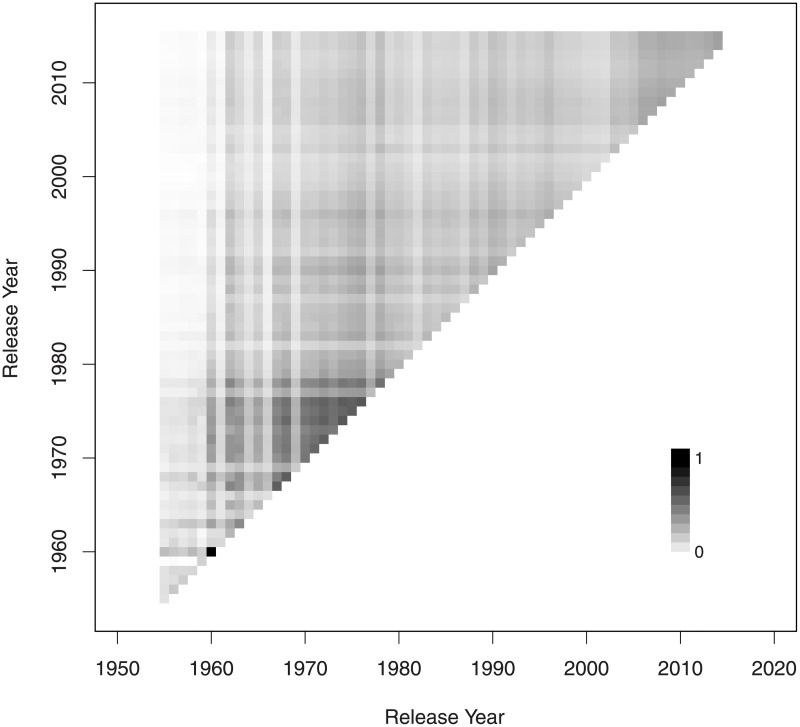
Interactions between the sets.

Because, smaller sets are likelier to be one-off, we have done the commonality analysis for all 10953 sets as well as for the 8848 sets with the size of at least 10. For the sets of all sizes, a total of 10278 (93.8%) shared at least one brick with at least one other set released in the same year, and a total of 10623 (97.0%) shared at least one brick with at least one other set released in the same year or later. For the sets of size 10 or more, the corresponding figures were 8731 (98.7%) and 8793 (99.4%) respectively. Fig 7 shows, for any pair of release years, the extent to which any two non-exclusive sets share pieces in common. The pattern is very similar for the sets of size 10 and above.

**Fig 8:**
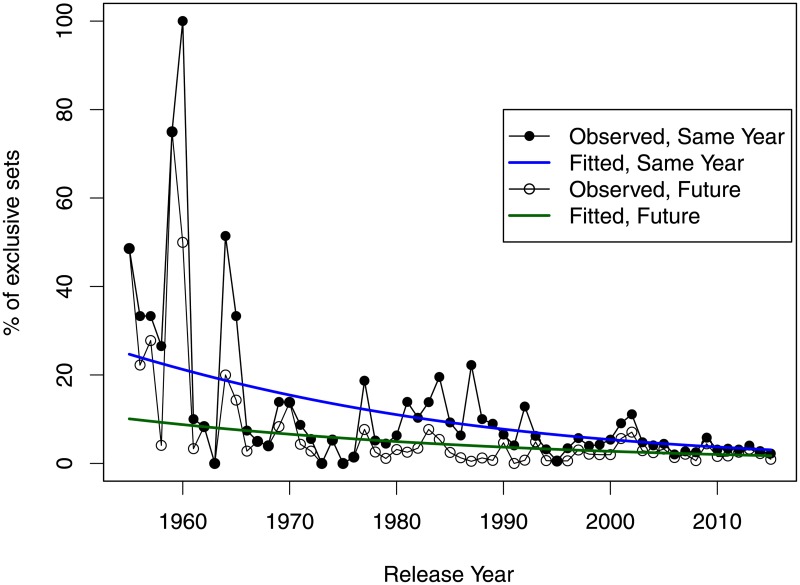
Proportion of all sets, which have no bricks in common with any sets released in the same year or at all. The pattern is similar for sets of size 10 and above (not shown).

The number of the ‘exclusive’ sets, which have no pieces in common with other sets is thus very small, and has been getting smaller. Fig 8 shows the evolution of the proportion of such exclusive sets. The odds of a set having no bricks in common with any sets released on the same year have been found to decrease at an average annual rate of 4% (95% CI: 3.5%; 4.5%, *p* < .0001) for all sets and at an average annual rate of 6.8% (95% CI: 5.6%; 8.0%, *p* < .0001) for the sets of size 10 and above. The odds of a set having no bricks in common with any sets released in the same year or later has been found to decrease at a slower rate for all sets, 3.1%, (95% CI: 2.4%; 3.8%, *p* < .0001), but at a faster rate of 8.7% for the sets of size 10 and above (95% CI: 6.9%; 10.5%, *p* < .0001). By the year 2015, the proportion of all such sets was found to be 2.7%.

**Fig 9:**
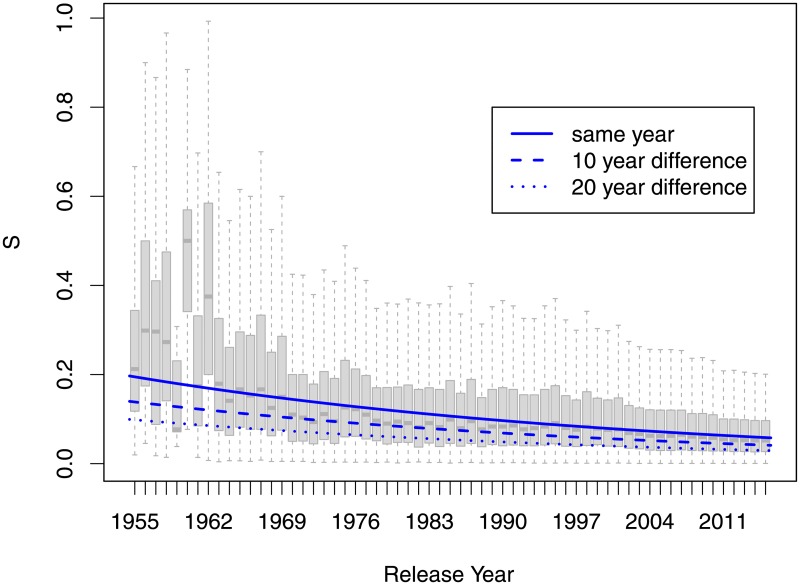
Index of commonality by release year.

For non-exclusive sets, the index of commonality was found to have decreased from an average 0.12 in 1955 to an average of 0.09 in 2015. This corresponded to an average estimated 2% annual decrease (*p* < 0.0001) as shown in Fig 9. The sets released closer together tended to have more in common. Each additional year between the sets was associated with an average 3.4% decrease in the commonality index *S*, *p* < .0001. The results were the same for the sets of size 10 and above.

## Conclusion

The number of bricks and sets that TLG produces each year has increased annually by around 7%. The size of the sets, meaning the number of bricks in each set, has also increased by an average of 1.9% per year while the number of bricks of the largest set in each year increased on average by a staggering 5.0%. There is also a significant shift towards larger sets per decade. Fig 5 shows how the number of bricks TLG offers per year has increased exponentially. We can clearly conclude that the sizes of sets has significantly increased which may also justify their price increase as it was already argued by [12].

The sets also include more diverse bricks. The average number of brick types in a set has increased on average by 2.4% per year and the maximum number of brick types in a set has even increased by 4.1%. The bricks have also become more specialized since their expected occurrence in the five year period following the sets release has decreased by 4.8% annually.

The sets have not only become larger and more diverse, they have also become more colorful. The number of colours in a set has been increasing at the average rate of 2.4% per year and the maximum number of colours in a set has been increasing at the average rate of 3.5% per year. Overall, the number of colours has increased exponentially at average annual rate of 4.4%.

Sets have also less parts in common. The commonality has decreased at an annual rate of 2.0% and is today at an absolute value of only 0.09. This is inline with the observation that the set sizes have increased and that the bricks have become more specialized.

Overall we can conclude that the LEGO products have indeed become far more complex. While TLG is still selling a product line of “basic” bricks, the number and sizes of sets has increased significantly. Sets have become more colorful and the bricks more specialized. Sets also share less and less bricks with each other. A judgment of whether this increase in complexity is good or bad remains difficult. Anybody interested in “basic” bricks can still purchase sets that only contain those. Nobody is forced into buying specialized bricks and sets. One can, however, argue that the strong reliance on more specialized bricks in combination with strong association to licensed themes may inhibit children to take their carefully build models apart to build something completely new. On the other hand one can argue that the bricks are comparable to a language. The increase in vocabulary size only means that writers can express themselves in ever more complex and imaginative ways. The gap between master builders and instruction followers may increase. But if we learned anything from The LEGO Movie [19] than it is that you need both, creativity and the ability to work in a team using instructions to build monumental designs.
